# The genetic vulnerability to cisplatin ototoxicity: a systematic review

**DOI:** 10.1038/s41598-019-40138-z

**Published:** 2019-03-05

**Authors:** Evangelia Tserga, Tara Nandwani, Niklas K. Edvall, Jan Bulla, Poulam Patel, Barbara Canlon, Christopher R. Cederroth, David M. Baguley

**Affiliations:** 1 Experimental Audiology, Biomedicum, Karolinska Institutet, Solnavägen 9, 171 65 Stockholm, Sweden; 2 School of Medicine, University of Nottingham, Nottingham, UK; 3 Department of Mathematics, University of Bergen, Bergen, Norway; 4 Department of Psychiatry and Psychotherapy, University Regensburg, Universitätsstraße 84, 93053 Regensburg, Germany; 5 Division of Oncology, School of Medicine, University of Nottingham, Nottingham, UK; 6 Otology and Hearing Group, Division of Clinical Neuroscience, School of Medicine, University of Nottingham, Nottingham, UK; 7 NIHR Nottingham Biomedical Research Centre, University of Nottingham, Nottingham, UK

## Abstract

Ototoxicity is one of the major side-effects of platinum-based chemotherapy, in particular cisplatin (cis-diammine dichloroplatinum II). To our knowledge, no systematic review has previously provided a quantitative summary estimate of the impact of genetics upon the risk of developing hearing loss. We searched Embase, Medline, ASSIA, Pubmed, Scopus, and Web of Science, for studies documenting the genetic risk of ototoxicity in patients with cancer treated with cisplatin. Titles/abstracts and full texts were reviewed for inclusion. Meta-analytic estimates of risk (Odds Ratio) from the pooled data were calculated for studies that have been repeated twice or more. The search identified 3891 papers, of which 30 were included. The majority were retrospective (44%), ranging from n = 39 to n = 317, some including only patients younger than 25 years of age (33%), and some on both genders (80%). The most common cancers involved were osteosarcoma (53%), neuroblastoma (37%), prostate (17%) and reproductive (10%). Most studies performed genotyping, though only 5 studies performed genome-wide association studies. Nineteen single-nucleotide polymorphisms (SNPs) from 15 genes were repeated more than twice. Meta-analysis of group data indicated that rs1872328 on *ACYP2*, which plays a role in calcium homeostasis, increases the risk of ototoxicity by 4.61 (95% CI: 3.04–7.02; N = 696, *p* < 0.0001) as well as *LRP2* rs4668123 shows a cumulated Odds Ratio of 3.53 (95% CI: 1.48–8.45; N = 118, *p* = 0.0059), which could not be evidenced in individual studies. Despite the evidence of heterogeneity across studies, these meta-analytic results from 30 studies are consistent with a view of a genetic predisposition to platinum-based chemotherapy mediated ototoxicity. These new findings are informative and encourage the genetic screening of cancer patients in order to identify patients with greater vulnerability of developing hearing loss, a condition having a potentially large impact on quality of life. More studies are needed, with larger sample size, in order to identify additional markers of ototoxic risk associated with platinum-based chemotherapy and investigate polygenic risks, where multiple markers may exacerbate the side-effects.

## Introduction

Early detection and modern treatments for cancer have contributed to improved survival rates for many types and sites of disease, such that there are presently 14.5 million cancer survivors in the United States alone^1^. As the number of cancer survivors increases, so does the need to understand and moderate the factors that may adversely impact quality of life in survivorhood. One such factor is that of hearing loss, which has been shown in general populations to have adverse consequences for cognition^2^, and mental health^3^, if untreated. Specifically, hearing loss is a significant risk factor for dementia^2,4^. Given the vulnerability of many cancer survivors, the understanding of ototoxicity arising from cancer therapies is of high importance.

Treatment with cisplatin (cis-diamminedichloroplatinum II or CDDP) chemotherapy continues to be a mainstay of curative therapy for many common cancers including breast, testis, and ovarian cancer in adults, and paediatric neuroblastoma. The propensity for cisplatin to instigate cochlear dysfunction, and hence deficits in auditory sensitivity and discrimination abilities has long been known^5^, although this is still not clearly communicated to the cancer patient in the current medical practice^4^. The prevalence of hearing loss following cisplatin treatment is dependent upon cumulative dose^6^, and has been reported as being up to 90%^7^. Given the recent classification of hearing problems as the 4th leading cause in years lived with disability by the WHO^8^, interventions that cause hearing problems as a side-effect can have significant adverse consequences for quality of life. Whilst cisplatin administration leads to changes in auditory function that are detectable during and immediately after treatment, specifically bilateral progressive and irreversible high frequency hearing loss, the long-term persistent presence of the platinum compounds in the cochlea^9,10^ can increase the vulnerability to subsequent insults (age related metabolic change, noise, or viral, for example) and cumulate towards greater social communication impediments and burden.

The anti-cancer actions of cisplatin are mainly due to its interference with tumour cell proliferation^11^. Through its binding to nuclear DNA, cisplatin blocks transcription and causes double-strand breaks leading to cell cycle arrest^12,13^. However, since cells from the cochlea do not proliferate, it is thought that platination of mitochondrial DNA is a more likely cause of hearing loss than nuclear DNA damage^14,15^. It is generally known that cisplatin ototoxicity has 3 major targets, hair cells, spiral ganglion neurons and the stria vascularis (the metabolic hub of the cochlea)^16,17^. Several molecular mechanisms have been described as mediators of cisplatin-induced ototoxicity. Cisplatin has been shown to target the NOX3 anti-oxidant system by causing the formation of reactive oxidative species (ROS), which in turn triggers inflammatory pathways in the cochlea and promotes apoptotic and necrotic cell death^18^. Downstream of ROS generation is the JNK pathway, which activates STAT-1 mediated inflammatory pathways, leading to the induction of apoptotic cascades involving caspase 3 and 9^19^. Elegant *in vitro* and *in vivo* animal experiments have recently evidenced the involvement of the ATM-Chk2-p53 signaling pathway in cisplatin-mediated hair cell damage^20^. Interestingly, the stria vascularis retains platinum-based compounds for a long period of time, leading to subsequent alterations in potassium homeostasis and in the generation of the endocochlear potential, both being essential for normal hearing function^9,21^. Consequently, strial pathology could potentially contribute in disruptions in cochlear metabolic balance, production of ROS, and subsequent apoptosis of cochlear hair cells.

Several risk factors for ototoxicity related to cisplatin chemotherapy have been identified, including poor renal function, very young or old age, gender, nutritional status, melanin content, and pre-existing cochlear hearing loss^22^. A genetic predisposition has also been proposed based upon observations of substantial inter-individual variability in the prevalence and severity of ototoxicity^23^. Whilst there are multiple potential pathways for ototoxic hearing loss associated with cisplatin, the possibility of genetic susceptibility to ototoxic side effects is of interest from a number of perspectives^24^. The identification of polymorphisms that render individuals vulnerable to chemotherapy induced hearing loss is an important precursive step to precision individualized medicine approach that might titrate a chemotherapy regimen such that hearing loss was less likely or severe. Further, such knowledge would support translational genomic approaches in this area^25^. Additionally, it has been suggested that ototoxicity may act as a valid surrogate marker for other, less well defined health tissue damage associated with platinum-based chemotherapy^26^.

The aim of the present study was to perform a systematic review and meta-analysis of the literature pertaining to potential genetic predisposition to ototoxicity associated with cisplatin chemotherapy in humans.

## Methods

### Search strategy

**Figure 1:**
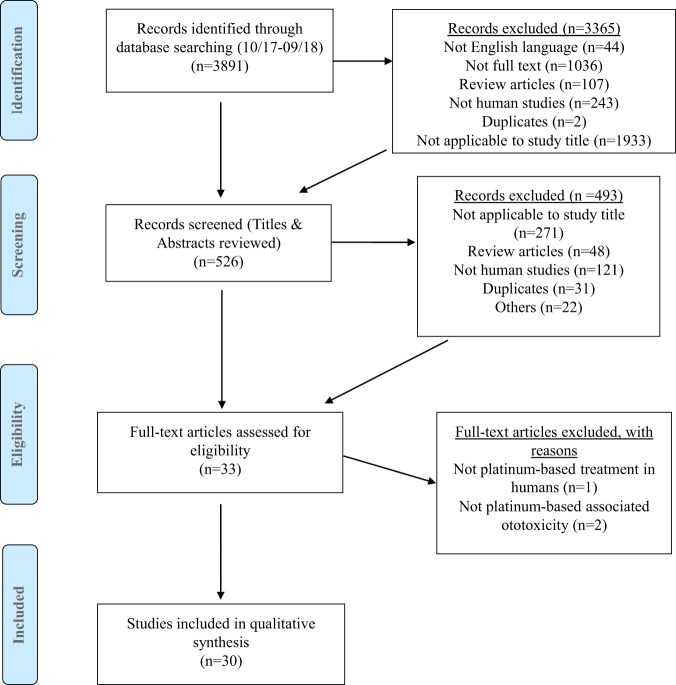
PRISMA flow diagram displaying the methodology used in the systematic review. The number of records identified by the search and the number of records excluded at each stage of screening against the inclusion/exclusion criteria is shown.

A systematic search of the literature was conducted by two of the authors (E. T. & T. N.) from 6 different databases: Embase, Medline, ASSIA, Pubmed, Scopus and Web of Science. For each database, the search was performed using the key terms: (Gene* OR genotype OR genetic) AND (tinnitus OR ototoxic* OR hearing loss OR hearing impairment OR hearing disorder OR cochleotoxicity OR deaf*) AND (Cisplatin OR cisplatinum OR platamin OR neoplatin OR cismaplat OR cis-diamminedichloridoplatinum* OR carboplatin OR paraplatin OR oxaliplatin OR (platinum AND chemotherapy). Literature searches were conducted in October 2017 and updated in September 2018 (Fig. 1**)**.

### Criteria for considering studies for this review

All studies written in English were considered eligible for this review. There was no restriction on participant age since studies with both children and adults were included. All different study designs were taken into account. Studies that were not available in English were excluded as we did not have the resources to translate them. Both adults and children were included in the review as many of the studies have been in children and since it is known that cisplatin causes more severe ototoxicity in children than in elders^22^. *In vitro* and *in vivo* studies were excluded because cell lines and animals are not fully representative of the ototoxic effects that platinum-based chemotherapy could have on humans.

### Data Extraction and Management

Data extracted included study design, demographic characteristics, intervention and genetic association. Data extraction tables were developed and piloted for this purpose. Where data were missing or unclearly reported, an attempt was made to contact the relevant corresponding author of the study. Three articles were excluded after reading the full text. One paper was excluded because platinum-based chemotherapy was only studied by *in vitro* methods^23^. Another was excluded because there was no association between cisplatin ototoxicity and the mitochondrial mutations, which they analysed and there is no report of ototoxicity grade^27^. A third paper was excluded because the statistical results are based on comparison with craniospinal radiation^28^. A study by *Upadhya et al*. reported that 31.4% of patients had sensorineural hearing loss 6 months after radiation of the ear^29^. Radiation causes ototoxic effects, therefore is a confounding factor when investigating the ototoxic effect of chemotherapy. Studies by Brown *et al*.^30^, Drögemöller *et al*.^31^, Olgun *et al*.^32^ and Wheeler *et al*.^33^ are included in the socio-demographic and the cisplatin intervention tables but not in the forest plots since not all values about patients with or without ototoxicity in relationship with the genetic profile were available.

The data from each article was extracted and summarized in an extraction form (Table 1 & Sup. Table S1). The extraction form includes socio-demographic data of the study participants, details of the treatment intervention and audiological assessment and the results of the statistical analysis of the genes examined. The Critical Appraisal Skills Program checklist was used to assess the validity and results of each article included in the systematic review (CASP Critical Appraisal Skills Program Oxford UK, 2017).

**Table 1:** Description of the socio-demographic data from the collected literature.

Demographic data, classified by year of publication					
Record	Study design	Sample size	Ethnicity	Median age (min, max)	Gender ratio (m/f)
Peters, 2000^38^	N/S	39	N/S	(3–22)	22/17
Peters, 2003^39^	N/S	39	N/S	(3–22)	23/16
Oldenburg, 2007^48^	retrospective	173	Norwegian	42 (24–73)	173/0
Oldenburg, 2007^49^	retrospective	238	Norwegian	29.3 (14.6–63.6)	238/0
Riedemann, 2008^40^	N/S	50	N/S	(5–22)	27/23
Barahmani, 2009^41^	N/S	42	Hispanic, Non-Hispanic white, African American, Other	6.8 (1.6–18)	34/8
Caronia, 2009^51^	retrospective	91	N/S	14.9 (3.7–34)	51/40
Ross, 2009^42^	case-control	162	Caucasian	7.5 (0–19)	99/63
Xu, 2012^52^	prospective	204	N/S	55 (33–77)	143/61
Choeyprasert, 2013^56^	case-control	68	Thai	N/S	40/28
Khokhrin, 2013^36^	N/S	87	Yakt Russian	N/S	0/87
Pussegoda, 2013^43^	case-control	317	Caucasian	8.5 (0–25)	77/78
Yang, 2013^44^	retrospective	213	White, Non White	(3.11–21.56)	141/72
Xu, 2013^61^	retrospective	282	Han Chinese	56 (34–76)	192/90
Hagleitner, 2014^57^	retrospective	148	Dutch, Spanish	(4–40)	76/72
Spracklen, 2014^59^	prospective	100	Caucasian, Cape mixed, Black African, Indian, Unknown	46.5 (14–75)	73/27
Brown, 2015^45^	N/S	71	Non-Hispanic white, Hispanic, Other	(0.7–18)	52/19
Lanvers-Kaminsky, 2015^37^	retrospective	64 pediatric & 66 adults	N/S	(5–22)	pediatric 38/ 26 adult 32/ 34
Xu, 2015^54^	retrospective	306	N/S	N/S	148/90
Olgun, 2016^32^	prospective	72	N/S	N/S	40/32
Talach, 2016^53^	prospective	55	N/S	35	52/3
Vos, 2016^58^	retrospective	156	Dutch	(3.4–43.9)	84/72
Brown, 2017^30^	retrospective	80	White, Hispanic, Other	(3.7–18.2)	57/23
Drögemöller, 2017^31^	retrospective	188	North American	31 (24–39)	188/0
Lopes-Aguiar, 2017^55^	prospective	90	Caucasian, IndigenousN/S	56 (27–74)	83/7
Spracklen, 2017^60^	N/S	222	African, Indian, mixed ancestry	48 (14–75)	158/64
Thiesen, 2017^46^	retrospective	116	White, Asian, African	(0–19)	74/42
Wheeler, 2017^33^	N/S	511	N/S	(18–55)	511/0
Drögemöller, 2018^50^	N/S	229	European, East Asian, South Asian, American, African	(23–49)	N/S
Lui, 2018^47^	retrospective	106	N/S	2.5 (0.2–16.9)	49/57

*N/S: Not specified.

### Risk of bias (Quality assessment)

Risk of bias assessment was conducted by four authors (E.T., T.N., N.E. & C. R. C) on those study records included in the meta-analysis. Risk of bias criteria that were taken into consideration in this review were the study population (age, gender, ethnicity), type of cancer (any type of malignancies), type of intervention (other ototoxic drugs, irradiation, prior hearing loss) and measurement of hearing outcome. All these criteria were taken into account in the interpretation of the results.

### Meta-analysis

Forest plots were created using the Forest Plot add-in (version 8.0) for JMP 13.2.1 data analysis software to visually summarize the results from each study included in this review. The forest plots display the odds ratios (OR) and 95% confidence intervals that demonstrate the association between ototoxicity and the various genes and single-nucleotide polymorphisms (SNPs) reported in the literature. One forest plot was created to demonstrate the results for the genes tested in a single study. A second forest plot was created to compare the findings of different studies examining the same genes and SNPs and provide an estimate of the combined result of these studies. This is a meta-analysis of the data. The combined odds ratios and 95% confidence intervals were calculated using the values for the number of variant SNPs and controls in the patient groups with normal hearing and with ototoxicity after chemotherapy.

### Statistical analysis

The number of cases from the included publications were extracted to four groups: Ototoxicity with SNP variant (OtSNP), Ototoxicity no SNP variant (Ot), Normal hearing with SNP variant (NhSNP), Normal hearing no SNP variant (Nh), and arranged the groups in a contingency table. Since the number of observations in all contingency tables considered is not too large, Fisher’s exact test serves for hypothesis testing. Although Fisher’s exact test is preferable whenever the computational power allows to carry it out, we also report results from the commonly used chi-squared (χ^2^) test to ensure comparability with the literature.

Furthermore, we report odds ratios (OR) for quantifying the risk of ototoxicity. The OR for being affected by ototoxicity if also having the SNP variant was then calculated as:

$$OR=\frac{OtSNP/NhSNP}{Ot/Nh}$$

Employing the Woolf method^34,35^, the 95% confidence interval (CI) of the odds ratio is given by:

$$95 \% \,CI=\exp (\mathrm{ln}(OR)\pm 1.96\,\times SE)$$ where $$SE=\,\sqrt{\frac{1}{NhSNP}+\frac{1}{Nh}+\frac{1}{OtSNP}+\frac{1}{Ot}}$$

For tables containing empty cells (i.e. if no research subjects populated a group), we applied the Haldane-Anscombe correction^36,37^, that is we added 0.5 to all cells in the table for that publication. The statistical procedures were carried out using JMP 13.2.1. Values of *p* < 0.05 were considered significant.

## Results

From the 30 included papers, 44% were retrospective with a sample size ranging from 39 to 317 (Table 1). Some of them (33%) were performed on patients younger than 25 years of age^30,37–47^ and 80% on both genders. The ethnicity was rather broad with Northern America and Norwegians representing together the majority of the articles (14%)^31,41,48–50^. However, 12 of the papers did not specify the ethnicity of the patients^32,33,37–40,47,51–55^ and 4 papers did not include age^32,36,54,56^, which is a known risk factor for ototoxicity^22^.

Supplementary Table S1 presents medical aspects reported in the studies. Fifty-three percent of the studies were performed on osteosarcoma^32,37–40,42–44,46,47,51,56–60^, medulloblastoma in 33%^30,32,38–41,44–46,56^, while testicular cancer was studied in 17% of them^31,33,48–50,53^. How the dose was reported varied between the studies with 17 studies reporting median values of cisplatin administration (from 100 to 525.5 mg/m^2^)^30,31,36,42–44,47–50,52,54–59^, and reporting mean values (from 328.2 to 425.5 mg/m^2^)^32,38,39,45^. Seven studies only reported the range^33,37,40,46,51,53,60^, and 3 the dose per cycle^41,60,61^. Only 9 studies reported the number of cycles/courses^31,41,44,48,51,52,54,59,61^. Regarding auditory measures, there was also a large heterogeneity. Information on the tests and metrics used was missing in 9 studies^36,41–43,51,52,55,58,61^. Sixty-three percent of the studies used pure-tone audiometry (PTA), of which 5 included auditory brainstem responses (ABR)^32,37,39,44,54^ and 4 included distortion products of otoacoustic emissions (DPOAE)^32,37,39,40^. However, 33% did not measure hearing at baseline, which makes the changes in hearing difficult to assess^30,33,42,43,48,49,51,55,56,58^. The average percent of patients with ototoxicity (>grade 2) was 41.8%, ranging from 8 to 75%. There were 6 different grading systems of ototoxicity used across the literature: Brock classification, CTCAE, Boston classification, Chang classification, ASHA, Muenster classification and the Standard National Cancer Institute classification. Nevertheless, in 10 papers there was no clarification of the ototoxicity grading system used^33,36,38,39,45,48–51,55^.

Radiotherapy or other ototoxic drugs (such as aminoglycosides and vincristine) were used as part of the treatment of patients in 24 of the included studies. Six papers did not specify whether other ototoxic drugs or radiotherapy were used^33,37–40,52^. Radiation to the head and neck causes ototoxic effects and is a confounding factor when investigating the ototoxic effect of chemotherapy^29^. Only 8 studies adjusted the statistical analysis to relevant clinical variables, such as age at diagnosis, gender, ethnic group, cumulative cisplatin dose, vincristine treatment and craniospinal irradiation doses^43,47–50,55,59,60^.

**Figure 2:**
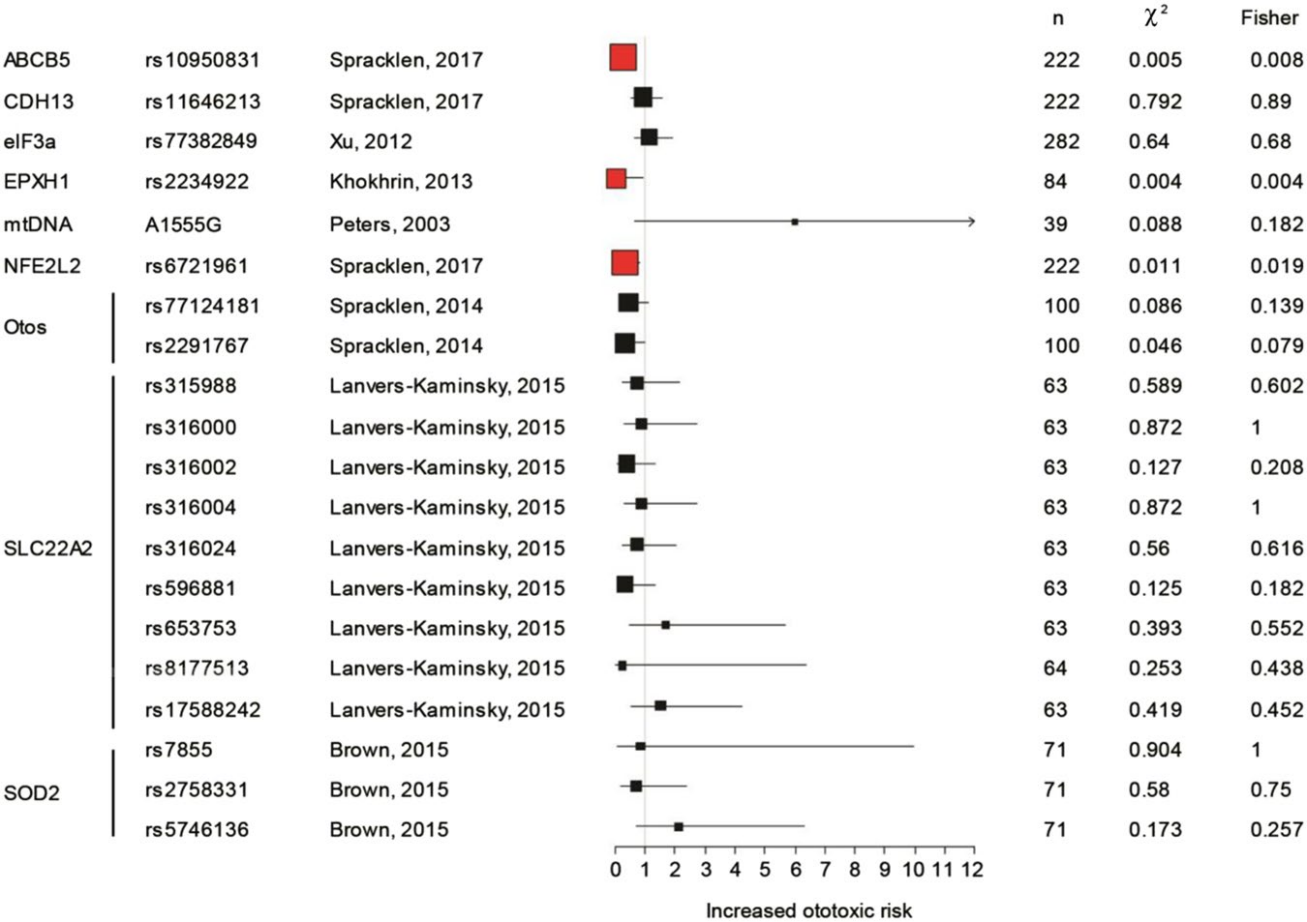
Forest plot describing the genes/SNPs tested in one study in alphabetic order. Black indicates a non-significant association with ototoxicity, red indicates a significant association with otoprotection, and blue indicates a significant association with ototoxicity. The square is centred on the odds ratio and the horizontal line represents the 95% confidence interval. n = sample size. The asterisk (*) identifies studies in which the p value to reach significance differed between Fishers and χ^2^ tests.

Twenty SNPs of 9 genes were investigated once without having been repeated (Fig. 2). Three of the SNPs were shown to be otoprotective^36,38,60^. Epoxides are among the many targets of GSTs. Converging this pathway, the Epoxide Hydrolase 1 (*EPXH1*) rs2234922 was related to otoprotection (OR: 0.05; 95% CI: 0.00–0.94; n = 84; *p* = 0.004)^36^. Spracklen *et al*. identified two SNPs predictors of cisplatin otoprotection^60^; rs6721961 of *NFE2L2* gene, involved in the protection of cells against oxidative stress (OR: 0.34; 95% CI: 0.15–0.81; n = 222; *p* = 0.019) and rs10950831 of *ABCB5* gene, which contributes on the cellular efflux of cisplatin (OR: 0.30; 95% CI: 0.12–0.73; n = 222; *p* = 0.008).

**Figure 3:**
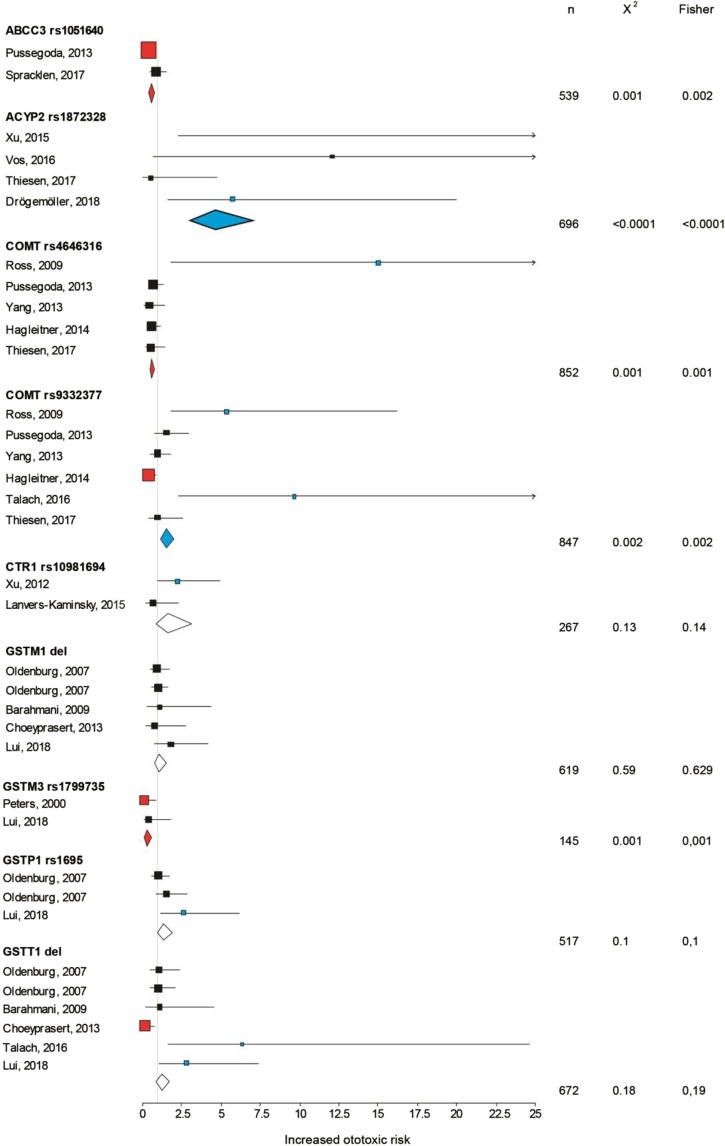
Forest plot describing *ABCC3* rs1051640, *ACYP2* rs1872328, *COMT* rs4646316, *COMT* rs9332377, *CTR1* rs10981694, *GSTM1* del, *GSTM3* rs1799735, *GSTP1* rs1695, *GSTT1* del tested in multiple studies. Black indicates a non-significant association with ototoxicity, blue indicates a significant association with ototoxicity and red a significant association with otoprotection. The square is centred on the odds ratio and the horizontal line represents the 95% confidence interval of each study. The diamond summarises each SNP average OR and the horizontal shows the 95% confidence interval. n = overall sample size. The asterisk (*) identifies studies in which the p value to reach significance differed between Fishers and χ^2^ tests.

**Figure 4:**
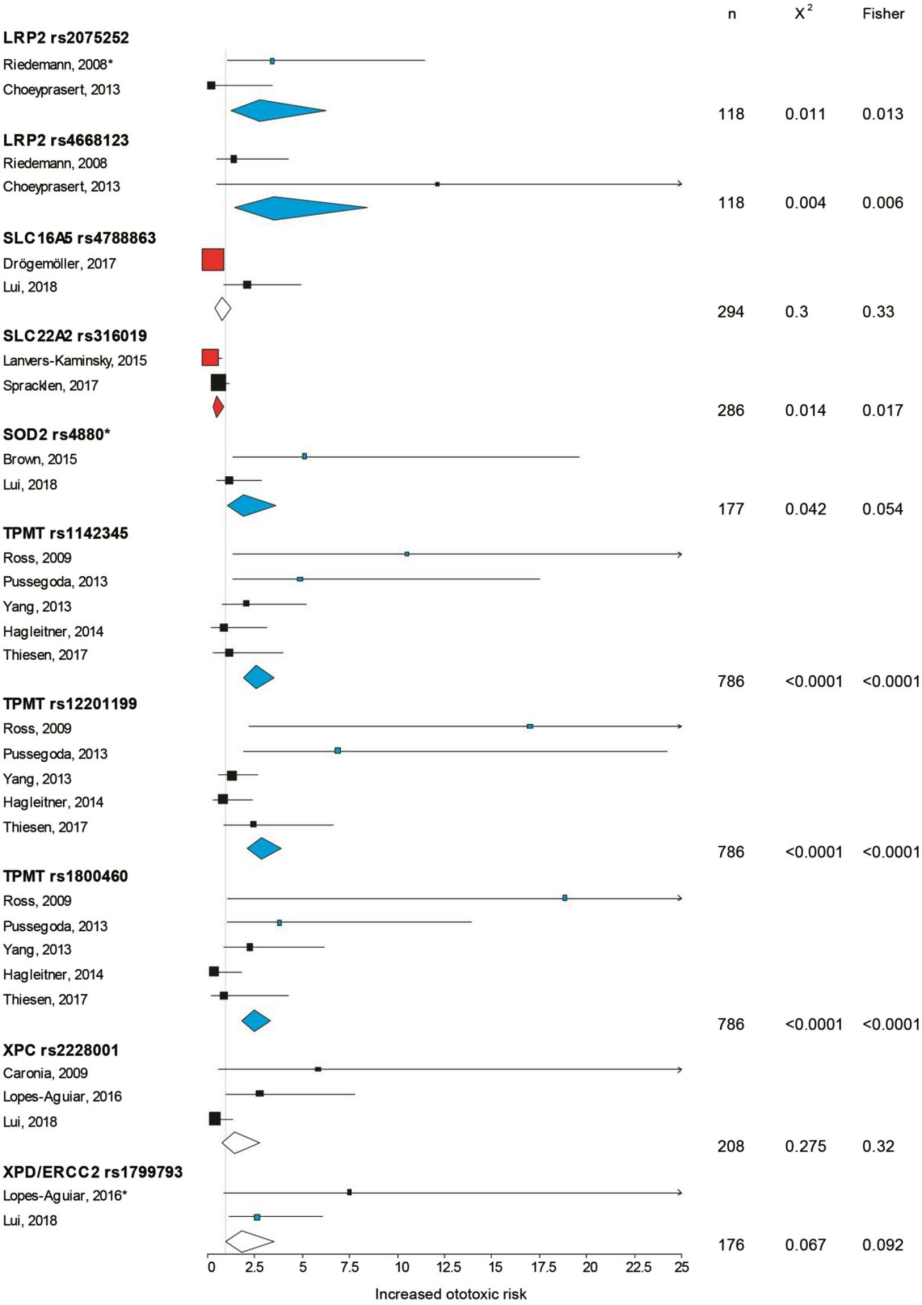
Forest plot describing *LRP2* rs2075252, *LRP2* rs4668123, *SLC16A5* rs4788863, *SLC22A2* rs316019, *SOD2* rs4880, *TPMT* rs1142345, *TPMT* rs12201199, *TPMT* rs1800460, *XPC* rs2228001, *XPD/ERCC2* rs1799793 tested in multiple studies. Black indicates a non-significant association with ototoxicity, blue indicates a significant association with ototoxicity and red a significant association with otoprotection. The square is centred on the odds ratio and the horizontal line represents the 95% confidence interval of each study. The diamond summarises each SNP average OR and the horizontal shows the 95% confidence interval. n = overall sample size. The asterisk (*) identifies studies in which the p value to reach significance differed between Fishers and χ^2^ tests.

Nineteen SNPs of 15 genes were investigated at least twice and the meta-analysis is shown in Figs 3 and 4. Seven of these SNPs showed no overall effect, namely the copper transport protein 1 *CTR1* rs10981694, *GSTM1* and *T1* deletions, *GSTP1* rs1695, *SLC16A5* rs4788863, *XPC* rs2228001 [a component of nucleotide excision repair (NER)] and *XPD* rs1799793. Albeit, *XPD* rs1799793 did not present an overall effect, was significantly ototoxic in one study (OR: 2.621; 95% CI: 1.13–6.10; n = 106; *p* = 0.034)^47^. The low-density lipoprotein-related protein 2 (*LRP2*) encoding the protein megalin was shown positively associated with ototoxicity on 2 SNPs (rs2075252 and rs4668123, Fig. 4). Interestingly, while the latter did not appear significant in hypothesis testing in 2 studies^40,56^, the accumulated data supports a positive association (OR: 3.532; 95% CI: 1.48–8.45; n = 118; *p* = 0.0059), likely due to an increase in the statistical power. Three SNPs (rs12201199, rs1142345, rs1800460) on the thiopurine S-methyltransferase gene (*TPMT*) were found significant in 2 studies^42,43^ and not in 3 others^44,46,57^. However, the overall pattern of the 5 studies merged together displayed significant associations with increased ototoxic risk from OR 2.47 to 2.82, with a total sample size of 786 (*p* < 0.0001). Another variant in *COMT* rs9332377 showed mixed results with 2 studies showing positive associations^42,53^, and 4 others not^43,44,46,57^, while Hagleitner *et al*. presented an otoprotective effect of this SNP (OR: 0.395; 95% CI; 0.19–0.83; n = 148; *p* = 0.014)^57^. The meta-analysis showed an overall positive association but with the smallest risk of all genes (OR: 1.55; 95% CI: 1.18–2.05; n = 847; *p* = 0.002). The acylphosphatase-2 *ACYP2* variant rs1872328, showed in Vos *et al*.^58^ and in Xu *et al*.^54^ reports a significant association (*p* = 0.0274 and *p* < 0.0001, respectively, by Fisher’s test), in spite of an extremely large confidence interval (Fig. 3). High OR of this SNP was also presented in another study showing the strong relation with cisplatin ototoxicity^50^. The combined data reveals a strong positive association with an overall risk of 4.618 (95% CI: 3.04–7.02; n = 696; *p* < 0.0001). Another gene playing an important role in oxidative stress, superoxide dismutase 2, mitochondrial (*SOD2*), with the rs4880 showing a positive association with cisplatin ototoxicity in one of the two studies^45^, but also in the overall meta-analysis with an OR of 1.917 (significant with χ^2^, but not with Fisher’s test, Fig. 4).

In contrast, some genes presented overall otoprotective associations in this meta-analysis such as the antioxidant polymorphism GSTM3*B (rs1799735) was associated with increased otoprotection (OR: 0.275; 95% CI: 0.13–0.59; n = 145; *p* = 0.001), a drug clearing transporter, namely the solute carrier *SLC22A2* rs316019 (OR: 0.485; 95% CI: 0.27–0.86; n = 286; *p* = 0.017) and rs1051640 of *ABCC3* gene (OR: 0.557; 95% CI: 0.39–0.798; n = 539; *p* = 0.0017). Although the *COMT* rs4646316 variant appeared ototoxic in one study^42^, no significant associations were found in the other studies^43,44,46,57^ and the overall meta-analysis instead presented otoprotective associations with an OR of 0.620 (*p* = 0.0008).

## Discussion

One of the most prevalent adverse effects of cisplatin treatment is ototoxicity, in which the consequent hearing loss - although is not lethal - has a non-negligible impact on life quality. Hearing deficits are now 4^th^ in the leading causes of years lived with disability^8^. It is thus an important factor to consider when performing cisplatin interventions in patients, not only to inform patients of the risks, but also to determine whether a given individual has a greater risk and for whom the regimen of administration may be adjusted or alternatives to cisplatin being given. The confirmation that there is a non-negligible risk for ototoxicity when treating patients with cisplatin, and that this risk is influenced by genetics, is in support for greater cautiousness in considering auditory impairments that cancer patients may develop.

A striking finding from this systematic review is that studies with non-significant findings in isolation reached sufficient power when combined to show increased risk of developing cisplatin-induced ototoxicity. This is the case for *LRP2* rs4668123, which emphasizes the need of considering larger sample sizes when performing such studies in order to provide more statistically solid evidence. Although our meta-analysis did not use individual data nor included adjustments (for instance for age, sex, the ethnic group, and the cumulative cisplatin dose), the summarized analysis emphasizes the need of large sample sizes to reveal biologically relevant associations that would otherwise been underestimated or missed.

We identified 8 different SNPs from 5 different genes (including rs4668123 from *LRP2*) from repeated studies showing significant associations with cisplatin ototoxicity (Table 2). These genes are mainly related to anti-oxidant regulation, neurotransmission or to auditory function. *ACYP2* encodes the acylphosphatase-2 expressed in the cochlea that hydrolyses phosphoenzyme intermediates of membrane pumps that affect Ca^2+^ ion homeostasis^54^. While ATP-dependent Ca^2+^ signalling has been shown to be involved in hair cell development, the exact role of *ACYP2* on the cochlea remains unknown^54^. Interestingly this *ACYP2* polymorphism showed the highest average risk (OR: 4.618), which suggests its major involvement in cisplatin ototoxicity and opens the possibility for more investigations addressing the contribution of this polymorphism in cisplatin-mediated ototoxicity.

**Table 2:** Summary of all ototoxic associations from repeated studies, listed from the greatest OR to the smallest.

Gene	SNP	OR	CI Low	CI High	Sample size	p (X^2^)	p of Fisher
ACYP2	rs1872328	4.618	3.04	7.02	696	<0.0001	<0.0001
LRP2	rs4668123	3.53	1.48	8.45	118	0.0026	0.0053
TPMT	rs12201199	2.822	2.06	3.86	786	<0.0001	<0.0001
LRP2	rs2075252	2.80	1.25	6.28	118	0.010	0.013
TPMT	rs1142345	2.618	1.93	3.56	786	<0.0001	<0.0001
TPMT	rs1800460	2.472	1.82	3.35	786	<0.0001	<0.0001
SOD2	rs4880	1.917	1.01	3.61	177	0.04	0.05
COMT	rs9332377	1.553	1.18	2.05	847	<0.0001	<0.0001

With an OR ranging from 2.8 to 3.53, the *LRP2* rs2075252 and rs4668123 polymorphisms also appear as important risk factors for developing cisplatin-mediated ototoxicity. *LRP2* or megalin is currently the only gene associated with Donnai-Barrow syndrome, a condition characterized by craniofacial anomalies, ocular abnormalities, sensorineural deafness and developmental delay^62^. *LRP2* is also connected with diabetic nephropathy, Lowe syndrome, Dent disease, Alzheimer’s disease (AD) and gallstone disease^63^.

TPMT is a methyltransferase, which enzymatic activity varies depending on polymorphisms of *TPMT* gene in chromosome 6^64^. A decreased enzymatic activity leads to myelosuppression, gastrointestinal intolerance, pancreatitis and hypersensitivity^64^. Here, 3 polymorphisms were found with significant OR (Table 2), ranging from 2.47 to 2.82 suggesting a strong involvement of *TPMT* in cisplatin-mediated ototoxicity. Again, 2 out of 5 studies found positive associations, and the overall outcome was a clear risk in spite of the 3 negative associations. Interestingly, rs12201199 has been linked to the 3A haplotype, leading to a reduced activity of the TPMT enzyme and greater toxicity of the anticancer drugs thiopurine and mercaptopurine^65^. It was recently demonstrated that HEI-OC1 and UB**/**OC-1 cells derived from the cochlea are more sensitive to cisplatin when expressing the TPMT*3A variant instead of the wild-type *Tpmt*^65^. In contrast, *Tpmt* knock-out mice do not display an increased sensitivity to cisplatin when administered at comparable levels as found in humans^66^, however this result might not be surprising given the known resistance of mice to cisplatin ototoxicity when compared to rats or guinea pigs^67^.

Of the two polymorphisms tested for *COMT*, only rs9332377 appeared as an important risk factor, although displaying the smallest OR of all validated studies (OR: 1.553). Mutations in *COMT* genes are implicated in sensorineural deafness. Hearing loss is less severe in subjects with *COMT* Met allele, possible due to the protective effect of dopamine on the hearing system^68,69^. While *COMT* has not been described in the cochlea, a homolog sharing 30% sequence identity, *Comt2* was found expressed in hair cells and mice homozygous for a missense mutation in *Comt2* showed sensorineural deafness due to degeneration of hair cells^70^. Overall, there are strong indications that catecholamines play a potential role in the auditory function. Thus, a greater vulnerability to cisplatin ototoxicity may arise when the function of the auditory system is already weakened.

Another important polymorphism related with increased risk of cisplatin ototoxicity is *SOD2* rs4880 presented an overall OR of 1.917. SOD2 catalyses the metabolism of the highly toxic superoxide anion to less but still toxic hydrogen peroxide. The SNP rs4880, which results in an exchange of valine against alanine, increases the catalytic activity of SOD2, leading to the accumulation of hydrogen peroxide and secondary ROS generation^45^. It is thus possible that altered mitochondrial function in the cochlea may increase the vulnerability to cisplatin ototoxicity. Notably, *SOD2* polymorphisms (IVS3-23T/G; IVS3-60T/G; and V16A) have also been implicated in noise induced hearing loss (NIHL)^71,72^.

Three polymorphisms, which have been evaluated twice, were found with a significant oto-protective effect, namely *ABCC3* rs1051640, *GSTM3* rs1799735 and *SLC22A2* rs316019. ABCC3 is an ATP-binding cassette member of the MRP subfamily which is involved in multi-drug resistance. This transporter regulates the efflux of organic anions, glutathione *S*‐conjugates and xenobiotics^73,74^. MRP expression in cancer cells correlates with resistance to cisplatin^75^. The mechanisms by which ABCC3 regulates cisplatin-induced hearing loss are unclear, but some studies suggest it may act upstream of GST^73^. Indeed, consistent with the otoprotective effects of the *ABCC3* variant, *GSTM3* rs1799735 shown to be otoprotective by Peters *et al*.^40^ but also in the overall analysis. *GSTM3* variations are indeed thought to alter the susceptibility to potential carcinogens and toxins^76^. SLC22A2 is a solute carrier that encodes CTR1, which is a plasma-membrane transport-protein that has an essential role in cisplatin uptake into cochlea hair cells. Since cisplatin accumulates in the stria vascularis from the cochlea^9^, polymorphism that positively affects monocarboxylate transporter function may improve the strial function affected by cisplatin.

Only two studies have evaluated polygenic effects on the vulnerability to cisplatin ototoxicity. Oldenburg *et al*. evaluated the cumulated risks of combinations in variants of *GSTT1*, *GSTM1* and *GSTP1*^48^. Such an approach makes sense when considering that the overall results for *GSTM1* and *T1* appeared inconclusive (Fig. 3). However, the combination of *GSTM1 null*, *T1 null* and *P1* Ile105/Ile105 alleles had a major impact on the risk for severe hearing impairment^48^. These findings are consistent with the known association of *GSTM1 null*, *T1 null* and *P1* Ile105/Ile105 genotypes with greater vulnerability of developing NIHL^77,78^ and an 8.88-fold increase in the risk of developing presbyacusis (sensorineural hearing loss caused by natural ageing)^79^. The incapacity of these individuals to conjugate certain metabolites may ultimately cause oxidative stress and damage to the cochlea, which would be exacerbated in presence of cisplatin. Pussegoda *et al*. also performed plurigenic analyses but included more complex models incorporating clinical and genetic variables^43^. Here the combination of *TPMT* rs12201199, *ABCC3* rs1051640, and *COMT* rs4646316 in a high risk group could reach an OR of 11 (95% CI: 3.2–37.6). Such studies highlight the need of considering multigenic screens when assessing the risk for ototoxicity which may be underestimated when considering a single marker.

There are a number of limitations to be noted in the present study. First, our meta-analysis was performed using group data and not individual data, which pre-empted the possibility of adjusting for e.g. age at diagnosis, gender, ethnic group, cumulative cisplatin dose. Second, in all studies reviewed, hearing loss associated with cisplatin chemotherapy was assessed immediately or soon after treatment. Given that cisplatin persists indefinitely in the human cochlea after such treatment^10^, the possibility of longer-term cochlear vulnerability (and hence progressive hearing loss) cannot be discounted. Third, ototoxic effects do not only lead to hearing loss, but also tinnitus, and vestibular toxicity^80^, which were not assessed in the present review, may help determining additional impacts on the auditory system that cannot be revealed with traditional audiometry. There are numerous ototoxicity grading scales used across the different studies. In the clinical trial setting, standardization is vital and the variability between different studies makes analysis more challenging^81^. Currently, there are 2 main categories of ototoxicity assessment criteria: (1) those measuring a change of hearing from baseline, such as the National Cancer Institute Common Terminology Criteria for Adverse Events (CTCAE), and (2) those measuring absolute hearing levels, such as the Brock or Chang classifications^81^. Interestingly, Spracklen *et al*. used the CTCAE and Chang grading scales, but also the American Speech-Language-Hearing Association criteria (ASHA), and the resulting associations differed depending on which scale is used^60^. As a matter of fact *NFE2L2* polymorphisms presented as significantly ototoxic in ASHA and CTCAE scales (the latter of which was included in Fig. 2) but not when Chang scale is used^60^. As a consequence, the selection of the grading scale can have a dramatic impact on the outcome of the study. These findings highlight the needs of determining the most sensitive measures in order to standardise the methodologies into the context of genetic testing in ototoxic vulnerability.

Finally, whilst a genetic predisposition to cisplatin mediated sensorineural hearing loss has been identified and may help identifying cancer patients with greater ototoxic risk, the specific mechanisms remain elusive. This would be an essential precursive step to the development of oto-protective therapy together with cisplatin interventions. It has recently been demonstrated that specifically targeting the p53 pathway protects from cisplatin ototoxicity while still maintaining cancer treatment efficacy^20^. Knowing the genetic predisposition to cisplatin is an important advancement for improving clinical treatment but now new therapies that target specific pathways are being developed to protect against cisplatin-induced ototoxicity.

## Supplementary information

Supplementary Table S1

## References

[1] Burstein HJ (2017). Clinical Cancer Advances 2017: Annual Report on Progress Against Cancer From the American Society of Clinical Oncology. J Clin Oncol.

[2] Thomson RS, Auduong P, Miller AT, Gurgel RK (2017). Hearing loss as a risk factor for dementia: A systematic review. Laryngoscope Investig Otolaryngol.

[3] Greenzang KA (2018). Hearing Loss. J Clin Oncol.

[4] Frisina RD (2016). Comprehensive Audiometric Analysis of Hearing Impairment and Tinnitus After Cisplatin-Based Chemotherapy in Survivors of Adult-Onset Cancer. J Clin Oncol.

[5] Lippman AJ, Helson C, Helson L, Krakoff IH (1973). Clinical trials of cis-diamminedichloroplatinum (NSC-119875). Cancer Chemother Rep.

[6] Landier W (2016). Ototoxicity and cancer therapy. Cancer.

[7] The L (2017). Hearing loss: time for sound action. Lancet.

[8] Wilson BS, Tucci DL, Merson MH, O'Donoghue GM (2017). Global hearing health care: new findings and perspectives. Lancet.

[9] Breglio AM (2017). Cisplatin is retained in the cochlea indefinitely following chemotherapy. Nat Commun.

[10] Sheth S, Mukherjea D, Rybak LP, Ramkumar V (2017). Mechanisms of Cisplatin-Induced Ototoxicity and Otoprotection. Front Cell Neurosci.

[11] Wang D, Lippard SJ (2005). Cellular processing of platinum anticancer drugs. Nat Rev Drug Discov.

[12] Siddik ZH (2003). Cisplatin: mode of cytotoxic action and molecular basis of resistance. Oncogene.

[13] Rybak LP, Ramkumar V (2007). Ototoxicity. Kidney Int.

[14] Hutchin TP, Cortopassi GA (2000). Mitochondrial defects and hearing loss. Cell Mol Life Sci.

[15] Lanvers-Kaminsky C, Ciarimboli G (2017). Pharmacogenetics of drug-induced ototoxicity caused by aminoglycosides and cisplatin. Pharmacogenomics.

[16] Schacht J, Talaska AE, Rybak LP (2012). Cisplatin and aminoglycoside antibiotics: hearing loss and its prevention. Anat Rec (Hoboken).

[17] van Ruijven MW, de Groot JC, Klis SF, Smoorenburg GF (2005). The cochlear targets of cisplatin: an electrophysiological and morphological time-sequence study. Hear Res.

[18] Rybak LP, Mukherjea D, Jajoo S, Ramkumar V (2009). Cisplatin ototoxicity and protection: clinical and experimental studies. Tohoku J Exp Med.

[19] Wang J (2004). Caspase inhibitors, but not c-Jun NH_2_-terminal kinase inhibitor treatment, prevent cisplatin-induced hearing loss. Cancer Res.

[20] Benkafadar N (2017). Reversible p53 inhibition prevents cisplatin ototoxicity without blocking chemotherapeutic efficacy. EMBO Mol Med.

[21] Thomas JP, Lautermann J, Liedert B, Seiler F, Thomale J (2006). High accumulation of platinum-DNA adducts in strial marginal cells of the cochlea is an early event in cisplatin but not carboplatin ototoxicity. Mol Pharmacol.

[22] Yancey A (2012). Risk factors for cisplatin-associated ototoxicity in pediatric oncology patients. Pediatr Blood Cancer.

[23] Grondin Y (2015). Genetic Polymorphisms Associated with Hearing Threshold Shift in Subjects during First Encounter with Occupational Impulse Noise. PLoS One.

[24] Gauvin DV, Yoder J, Zimmermann ZJ, Tapp R (2018). Ototoxicity: The Radical Drum Beat and Rhythm of Cochlear Hair Cell Life and Death. Int J Toxicol.

[25] Travis, L. B. *et al*. Chemotherapy-induced peripheral neurotoxicity and ototoxicity: new paradigms for translational genomics. *J Natl Cancer Inst* **106**, 10.1093/jnci/dju044 (2014). 10.1093/jnci/dju044 PMC4568989 24623533

[26] Oldenburg J, Gietema JA (2016). The Sound of Silence: A Proxy for Platinum Toxicity. J Clin Oncol.

[27] Knoll C, Smith RJ, Shores C, Blatt J (2006). Hearing genes and cisplatin deafness: a pilot study. Laryngoscope.

[28] Rednam S, Scheurer ME, Adesina A, Lau CC, Okcu MF (2013). Glutathione S-transferase P1 single nucleotide polymorphism predicts permanent ototoxicity in children with medulloblastoma. Pediatr Blood Cancer.

[29] Upadhya I, Jariwala N, Datar J (2011). Ototoxic effects of irradiation. Indian J Otolaryngol Head Neck Surg.

[30] Brown AL (2017). DNA methylation of a novel PAK4 locus influences ototoxicity susceptibility following cisplatin and radiation therapy for pediatric embryonal tumors. Neuro Oncol.

[31] Drogemoller BI (2017). Association Between SLC16A5 Genetic Variation and Cisplatin-Induced Ototoxic Effects in Adult Patients With Testicular Cancer. JAMA Oncol.

[32] Olgun Y (2016). Analysis of genetic and non genetic risk factors for cisplatin ototoxicity in pediatric patients. Int J Pediatr Otorhinolaryngol.

[33] Wheeler HE (2017). Variants in WFS1 and Other Mendelian Deafness Genes Are Associated with Cisplatin-Associated Ototoxicity. Clin Cancer Res.

[34] Ruxton, G. D. & Neuhäuser, M. Review of alternative approaches to calculation of a confidence interval for the odds ratio of a 2 × 2 contingency table. *Methods in Ecology and Evolution*, 9–13 (2013).

[35] Lawson R (2004). Small sample confidence intervals for the Odds Ratio. Communications in Statistics - Simulation and Computation.

[36] capital Ka, C. D. V. *et al*. Pharmacogenomics of cisplatin-based chemotherapy in ovarian cancer patients from Yakutia. *Mol Gen Mikrobiol Virusol* 6–9 (2013). 24645271

[37] Lanvers-Kaminsky C (2015). Human OCT2 variant c.808G>T confers protection effect against cisplatin-induced ototoxicity. Pharmacogenomics.

[38] Peters U (2000). Glutathione S-transferase genetic polymorphisms and individual sensitivity to the ototoxic effect of cisplatin. Anticancer Drugs.

[39] Peters U, Preisler-Adams S, Lanvers-Kaminsky C, Jurgens H, Lamprecht-Dinnesen A (2003). Sequence variations of mitochondrial DNA and individual sensitivity to the ototoxic effect of cisplatin. Anticancer Res.

[40] Riedemann L (2008). Megalin genetic polymorphisms and individual sensitivity to the ototoxic effect of cisplatin. Pharmacogenomics J.

[41] Barahmani N (2009). Glutathione S-transferase M1 and T1 polymorphisms may predict adverse effects after therapy in children with medulloblastoma. Neuro Oncol.

[42] Ross CJ (2009). Genetic variants in TPMT and COMT are associated with hearing loss in children receiving cisplatin chemotherapy. Nat Genet.

[43] Pussegoda K (2013). Replication of TPMT and ABCC3 genetic variants highly associated with cisplatin-induced hearing loss in children. Clin Pharmacol Ther.

[44] Yang JJ (2013). The role of inherited TPMT and COMT genetic variation in cisplatin-induced ototoxicity in children with cancer. Clin Pharmacol Ther.

[45] Brown AL (2015). SOD2 genetic variant associated with treatment-related ototoxicity in cisplatin-treated pediatric medulloblastoma. Cancer Med.

[46] Thiesen S (2017). TPMT, COMT and ACYP2 genetic variants in paediatric cancer patients with cisplatin-induced ototoxicity. Pharmacogenet Genomics.

[47] Lui G (2018). Association between genetic polymorphisms and platinum-induced ototoxicity in children. Oncotarget.

[48] Oldenburg J, Kraggerud SM, Cvancarova M, Lothe RA, Fossa SD (2007). Cisplatin-induced long-term hearing impairment is associated with specific glutathione s-transferase genotypes in testicular cancer survivors. J Clin Oncol.

[49] Oldenburg J (2007). Association between long-term neuro-toxicities in testicular cancer survivors and polymorphisms in glutathione-s-transferase-P1 and -M1, a retrospective cross sectional study. J Transl Med.

[50] Drogemoller BI (2018). Further Investigation of the Role of ACYP2 and WFS1 Pharmacogenomic Variants in the Development of Cisplatin-Induced Ototoxicity in Testicular Cancer Patients. Clin Cancer Res.

[51] Caronia D (2009). Common variations in ERCC2 are associated with response to cisplatin chemotherapy and clinical outcome in osteosarcoma patients. Pharmacogenomics J.

[52] Xu X (2012). Prediction of copper transport protein 1 (CTR1) genotype on severe cisplatin induced toxicity in non-small cell lung cancer (NSCLC) patients. Lung Cancer.

[53] Talach T (2016). Genetic risk factors of cisplatin induced ototoxicity in adult patients. Neoplasma.

[54] Xu H (2015). Common variants in ACYP2 influence susceptibility to cisplatin-induced hearing loss. Nat Genet.

[55] Lopes-Aguiar L (2017). XPD c.934G>A polymorphism of nucleotide excision repair pathway in outcome of head and neck squamous cell carcinoma patients treated with cisplatin chemoradiation. Oncotarget.

[56] Choeyprasert W (2013). Cisplatin-induced ototoxicity in pediatric solid tumors: the role of glutathione S-transferases and megalin genetic polymorphisms. J Pediatr Hematol Oncol.

[57] Hagleitner MM (2014). Influence of genetic variants in TPMT and COMT associated with cisplatin induced hearing loss in patients with cancer: two new cohorts and a meta-analysis reveal significant heterogeneity between cohorts. PLoS One.

[58] Vos HI (2016). Replication of a genetic variant in ACYP2 associated with cisplatin-induced hearing loss in patients with osteosarcoma. Pharmacogenet Genomics.

[59] Spracklen TF (2014). Genetic variation in Otos is associated with cisplatin-induced ototoxicity. Pharmacogenomics.

[60] Spracklen TF, Vorster AA, Ramma L, Dalvie S, Ramesar RS (2017). Promoter region variation in NFE2L2 influences susceptibility to ototoxicity in patients exposed to high cumulative doses of cisplatin. Pharmacogenomics J.

[61] Xu X (2013). Association between eIF3alpha polymorphism and severe toxicity caused by platinum-based chemotherapy in non-small cell lung cancer patients. Br J Clin Pharmacol.

[62] Li Q (2018). Megalin mediates plasma membrane to mitochondria cross-talk and regulates mitochondrial metabolism. Cell Mol Life Sci.

[63] Marzolo MP, Farfan P (2011). New insights into the roles of megalin/LRP2 and the regulation of its functional expression. Biol Res.

[64] Asadov C, Aliyeva G, Mustafayeva K (2017). Thiopurine S-Methyltransferase as a Pharmacogenetic Biomarker: Significance of Testing and Review of Major Methods. Cardiovasc Hematol Agents Med Chem.

[65] Bhavsar AP (2017). Pharmacogenetic variants in TPMT alter cellular responses to cisplatin in inner ear cell lines. PLoS One.

[66] Liu C (2017). Differential effects of thiopurine methyltransferase (TPMT) and multidrug resistance-associated protein gene 4 (MRP4) on mercaptopurine toxicity. Cancer Chemother Pharmacol.

[67] Poirrier AL (2010). Ototoxic drugs: difference in sensitivity between mice and guinea pigs. Toxicol Lett.

[68] Toro C (2015). Dopamine Modulates the Activity of Sensory Hair Cells. J Neurosci.

[69] Niu X, Canlon B (2006). The signal transduction pathway for the dopamine D1 receptor in the guinea-pig cochlea. Neuroscience.

[70] Du X (2008). A catechol-O-methyltransferase that is essential for auditory function in mice and humans. Proc Natl Acad Sci USA.

[71] Fortunato G (2004). Paraoxonase and superoxide dismutase gene polymorphisms and noise-induced hearing loss. Clin Chem.

[72] Liu YM (2010). SOD2 V16A SNP in the mitochondrial targeting sequence is associated with noise induced hearing loss in Chinese workers. Dis Markers.

[73] Ballatori N, Hammond CL, Cunningham JB, Krance SM, Marchan R (2005). Molecular mechanisms of reduced glutathione transport: role of the MRP/CFTR/ABCC and OATP/SLC21A families of membrane proteins. Toxicol Appl Pharmacol.

[74] Young LC (1999). Expression of multidrug resistance protein-related genes in lung cancer: correlation with drug response. Clin Cancer Res.

[75] Oguri T, Isobe T, Fujitaka K, Ishikawa N, Kohno N (2001). Association between expression of the MRP3 gene and exposure to platinum drugs in lung cancer. Int J Cancer.

[76] Checa-Rojas A (2018). GSTM3 and GSTP1: novel players driving tumor progression in cervical cancer. Oncotarget.

[77] Lin CY (2009). Glutathione S-transferase M1, T1, and P1 polymorphisms as susceptibility factors for noise-induced temporary threshold shift. Hear Res.

[78] Shen H (2012). Genetic variation in GSTM1 is associated with susceptibility to noise-induced hearing loss in a Chinese population. J Occup Environ Med.

[79] Manche SK, Jangala M, Putta P, Koralla RM, Akka J (2016). Association of oxidative stress gene polymorphisms with presbycusis. Gene.

[80] Callejo A (2017). Dose-dependent cochlear and vestibular toxicity of trans-tympanic cisplatin in the rat. Neurotoxicology.

[81] King, K. A. & Brewer, C. C. Clinical trials, ototoxicity grading scales and the audiologist's role in therapeutic decision making. *Int J Audiol*, 1–10, 10.1080/14992027.2017.1417644 (2017). 10.1080/14992027.2017.1417644 PMC6260812 29276851

[82] Schlee W (2017). Innovations in Doctoral Training and Research on Tinnitus: The European School on Interdisciplinary Tinnitus Research (ESIT) Perspective. Front Aging Neurosci.

