# Supplementary material for: The genetic vulnerability to cisplatin ototoxicity: a systematic review
Source: Sci Rep. 2019 Mar 5;9:3455. doi: 10.1038/s41598-019-40138-z (PMC6401165; doi:10.1038/s41598-019-40138-z)
Supplement: Supplementary file 1 — Supplementary Table S1 [file 41598_2019_40138_MOESM1_ESM.docx]

**The genetic vulnerability to cisplatin ototoxicity: a systematic review**

Evangelia Tserga*^1^, Tara Nandwani*^2^, Niklas K. Edvall^1^, Jan Bulla^3, 4^, Poulam Patel^5^, Barbara Canlon^1^, Christopher R. Cederroth^1^, David M. Baguley ^6, 7^

***Affiliations***

^1^ Experimental Audiology, Biomedicum, Karolinska Institutet, Solnavägen 9, 171 65 Stockholm, Sweden

^2^ School of Medicine, University of Nottingham, Nottingham UK

^3^ Department of Mathematics, University of Bergen, Norway

^4^ Department of Psychiatry and Psychotherapy, University Regensburg

Universitätsstraße 84 93053 Regensburg, Germany

^5^ Division of Oncology, School of Medicine, University of Nottingham, Nottingham UK

^6^ Otology and Hearing Group, Division of Clinical Neuroscience, School of Medicine, University of Nottingham, Nottingham UK

^7^ NIHR Nottingham Biomedical Research Centre, University of Nottingham, Nottingham UK

* Contributed equally

**Corresponding author**

Prof David Baguley,

NIHR Biomedical Research Centre,

Ropewalk House, 113 Ropewalk

Nottingham, NG1 5DU UK

Email: David.baguley@nottingham.ac.uk

**Supplementary Table S1. Description of the cisplatin interventions from the collected literature.**

| Os = osteosarcoma; GcT = Germ cell Tumor; Nb = Neuroblastoma; Mb = Medulloblastoma; Npc = Nasopharyngeal carcinoma; Hb = Hepatoblastoma; Rb = Retinoblastoma; Tc = Testicular cancer; Bt = Brain tumor; NSCLC = Non-small cell lung carcinoma; Sarc = any type of sarcoma | | | | | | | | | | | |  |
| --- | --- | --- | --- | --- | --- | --- | --- | --- | --- | --- | --- | --- |
| **Record** | **Tumour types** | **Sample size** | **Type of platinum therapy** | **Cumulative dose (mg/m2) M (median); m (mean)** | **Number of cycles/ courses** | **Duration (months)** | **Radiotherapy/ other ototoxic drugs** | **Audiological measures** | **at baseline / follow up** | **% patients (ototoxicity grade)** | **Scale** | |
| ***Genetic studies on cisplatin ototoxicity classified by decreasing dose*** | | | | | | | | | | | | |
| Choeyprasert, 2013^56^ | 26.5% Os; 27.9% GcT; 17.6% Nb; 11.8% Mb; 2.9% Npc; 2.9% Hb; 7.3% Sarc; 1.5% Glioblastoma multiforme, 1.5% Oligodendroglioma | 68 | Cisplatin | 525.5 (M) |  |  | craniospinal irradiation, aminoglycosides | Tymp, air & bone PTA (2.5-8 kHz), play PTA | no/yes | 67.6% (2) | Brock | |
| Hagleitner, 2014^57^ | Os | 148 | Cisplatin | 500 (M) |  |  | vincristine, vancomycin, gentamicin, tobramycin | conventional & play PTA | yes/yes | 39.18% (2-4) | CTCAE & Boston | |
| Vos, 2016^58^ | Os | 156 | Cisplatin | 480 (M) |  | 1-311 | vincristine, carboplatin | audiological assessments N/S, play PTA | no/yes | 23.1% (≥2a) | Chang | |
| Peters, 2000^38^ | 69.2% Os; 7.69% GcT; 15.38% Nb; 7.69% Mb. | 39 | Cisplatin | 425.5 (m) |  |  | N/S | PTA | yes/yes | 16.9% (3) | N/S | |
| Peters, 2003^39^ | 72% Os; 7.69% GcT; 12.82% Nb; 7.69% Mb | 39 | Cisplatin | 425.5 (m) |  |  | N/S | PTA, Tymp, DPOAE, ABR | yes/yes | 30.7% (3) | N/S | |
| Oldenburg, 2007^48^ | Tc | 173 | Cisplatin | 402 (M) | 3-4 |  | bleomycin, etoposide, vinblastine | air & bone PTA(2.5-8 kHz) | no/yes | N/S | N/S | |
| Olgun, 2016^32^ | 37.5% Nb; 15.2% Hb;15.2% GcT; 9.7% Npc; 5.5% Os; 2.7% Langerhans cell histiocytosis, 8.3% Mb; 2.6% Sarc; 2.7% Malignant melanoma | 72 | Cisplatin | > 400 (m) |  | 26 | radiotherapy to Head/Neck, furosemide, carboplatin, aminoglycosides | PTA, DPOAE, 6kHz & 8kHz tone burst ABRs | yes/yes | 30%/41.6% | Brock/Muenster | |
| Drögemöller, 2017^31^ | Tc | 188 | Cisplatin | 400 (M) | 4 |  | cranial irradiation/tobramycin, vancomycin, vincristine, furosemide | audiogram measurements (1-8 kHz) | yes/yes | 75.6% (3) | CTCAE | |
| Drögemöller, 2018^50^ | Tc | 229 | Cisplatin | 400 (M) |  |  | cranial irradiation, BEP, EP, VIP2 | audiogram measurements (0.25-8 kHz), geometric calculations of hearing thresholds (4, 6 & 8 kHz) | yes/yes | 16% (moderate-severe) | N/S | |
| Lui, 2018^47^ | 27.35% Nb; 17.92% Hb; 8.49% Rb; 34.9% Malignant germinal tumor, 11.32% Os | 106 | Cisplatin/ Carboplatin | cisplatin: 400 (M) carboplatin: 1518 (M) |  | 24-108 | aminoside, glycopeptide, furosemide | PTA, visual reinforcement audiometry | yes/yes | 31% (≥ 2) | Brock | |
| Pussegoda, 2013^43^ | 22% Bt; 0.63% Endodermal sinus tumor of thymus, 12.61% GcT; 16% Hb;1.26% Lymphoma, 0.94% Npc; 22.39% Nb; 22% Os; 0.31% Rb; 0.31% Mesenchymal tumor of the liver, 0.94% Other sarcoma, 0.31% Other carcinoma | 317 | Cisplatin | 400 (M) |  | 0-15 | cranial irradiation, vincristine | audiological assessments (details N/S) | no/yes | (2.71) | CTCAE | |
| Ross, 2009^42^ | 20.37% Bt; 0.61% Endodermal sinus, 13.5% GcT; 16.6% Hb; 0.61% Lymphoma, 0.61% Npc; 21.6% Nb; 24.69% Os; 1.23% Sarc | 162 | Cisplatin | 400 (M) |  | 1-12 | cranial irradiation, vincristine | N/S | no/yes | 63.8% (2-4) | CTCAE | |
| Oldenburg, 2007^49^ | Tc | 238 | Cisplatin | 397 (M) |  | 144 | bleomycin, etoposide, vinblastine | QLQ questionnaire module | no/yes | N/S | N/S | |
| Brown, 2017^30^ | 87.6% Mb; 12.4% PNET | 80 | Cisplatin | 330 (M) |  | 82.8 | craniospinal irradiation | air & bone PTA | no/yes | 21.25% (4) | Boston | |
| Brown, 2015^45^ | 93.8% Mb; 6.17% PNET | 71 | Cisplatin | 328.2 (m) |  |  | craniospinal irradiation | PTA, cochlear radiation information | yes/yes | 0.37 | N/S | |
| Yang, 2013^44^ | Mb, Nb & Os (percentage N/S) | 213 | Cisplatin | 300 (M) | 4 |  | craniospinal irradiation for Bt | conventional PTA (0.25-8 kHz), play PTA, visual reinforcement PTA or ABR | yes/yes | 70% (>0) | CTCAE & Chang | |
| Xu, 2015^54^ | Bt | 306 | Cisplatin + carboplatin | 287.6 (M) | 4 |  | craniospinal irradiation, vinblastine | conventional PTA (0.25-8 kHz), play PTA, visual reinforcement PTA or ABR | yes/yes | 37% (≥2a) | Chang | |
| Spracklen, 2014^59^ | 47% Head/Neck, 17% Reproductive, 2% Urological carcinoma, 16% Lymphoma, 10% Os; 6% Digestive, 2% Mixed | 100 | Cisplatin | 270 (M) | 1-6 |  | concurrent cranial irradiation, BEP, ondansetron | air & bone PTA (0.5-8 kHz) | yes/yes | 8% (3) | Chang | |
| Lopes-Aguiar, 2017^55^ | Head/Neck | 90 | Cisplatin | 265 (M) |  | 3.3-48.9 | Gy radiotherapy, ondansetron | N/S | no/yes | 36.6% (2-5) | N/S | |
| Khokhrin, 2013^36^ | Ovarian | 87 | Cisplatin | 100 (M) |  | 4.5 | cyclophosphamide | N/S |  | 5.7% (4) | N/S | |
| Xu, 2012^52^ | NSCLC | 204 | Cisplatin | 100 (M) | 2-6 (21 days/cycle), 1 week rest among the cycles |  | N/S | N/S |  | 43.1% | Standard National Cancer Institution | |
| Spracklen, 2017^60^ | 50.4% Head/Neck, 4.5% Oesophageal, 4.9% Gastric, 5.85% Os; 4.5% Lymphoma, 5.4% Other | 222 | Cisplatin | > 60 (M) |  | 36 | ondansetron | air & bone PTA (0.25-8 kHz), audiometry tests (GSI TympStar clinical middle-ear analyser, GSI 61 audiometer), audiograms | yes/yes | ASHA: 66.7% (1) Chang: 39.3% (>0) CTCAE: 57.7% (>0) | ASHA, Chang & CTCAE | |
| ***Genetic studies on cisplatin ototoxicity classified by decreasing dose - only range provided*** | | | | | | | | | | | | |
| Caronia, 2009^51^ | Os | 91 | Cisplatin | 120-1131 | 3 courses for 3 days each, maximum 4 weeks | 1 | methotrexate | objective audiometric tests (details N/S) | no/yes | N/S | N/S | |
| Thiesen, 2017^46^ | 30% Mb; 12.5% Hb; 24.2% Os; 12.5% Nb; 15.8% other CNS tumors, 5% other non-CNS tumors | 116 | Cisplatin + carboplatin | 60-800 |  |  | cranial irradiation | PTA (1-8 kHz) & visual response | yes/yes | 75.8%/71.4% | CTCAE & Chang | |
| Lanvers-Kaminsky, 2015^37^ | pediatric: 64% Os; 6.25% Nb; 18.75% Bt; 10.94% GcT; adult: Solid | pediatric:64 adult:66 | cisplatin | pediatric: 120-640 adult: up to 100 i.v |  | pediatric: 3-6.7 adult: 1-3 | N/S | Tymp, air & bone PTA, TEOAE & DPOAE, ABR | yes/yes | pediatric: 56.25% (>=1) adult: 27.27% (>=1) | pediatric: Muenster adult: NCI-CTCAE | |
| Wheeler, 2017^33^ | Tc | 511 | Cisplatin | < 300 or > 300 |  | 216 | N/S | air & bone PTA (4-12 kHz) | no/yes | N/S | N/S | |
| Riedemann, 2008^40^ | 76% Os; 6% Nb; 10% Mb; 4% GcT; 2% Teratoma, 2% Tc | 50 | Cisplatin | 180-300 |  | 151.2 | N/S | Tymp, air & bone PTA, TEOAE & DPOAE, ABR | yes/yes | 50% (≥ 2) | Muenster | |
| Talach, 2016^53^ | Tc, N/S for females | 55 | Cisplatin | 80-100 |  | 5.2 | etoposide, furosemide, bleomycin, vincristine, carboplatin | PTA (0.125-12 kHz) | yes/yes | N/S | Muenster | |
| Barahmani, 2009^41^ | Mb | 42 | Cisplatin | 75/cycle | 8 |  | craniospinal irradiation | audiological assessments (details N/S) | yes/yes | 56% (≥ 3) | CTCAE | |
| Xu, 2013^61^ | NSCLC | 282 | Cisplatin + carboplatin | 80/cycle | 2-6 |  | etoposide, gemcitabine, docetaxel | N/S |  | 50.35% (1-4) | Standard National Cancer Institution | |
| Spracklen, 2017^60^ | 14.8% Reproductive | 222 | Cisplatin | 25-60 | 3 days/cycle | 36 | etoposide, bleomycin | air & bone PTA (0.25-8 kHz), audiometry tests (GSI TympStar clinical middle-ear analyser, GSI 61 audiometer), audiograms | yes/yes | ASHA: 66.7% (1) Chang: 39.3% (>0) CTCAE: 57.7% (>0) | ASHA, Chang & CTCAE | |
